# Encoder–Decoder Variant Analysis for Semantic Segmentation of Gastrointestinal Tract Using UW-Madison Dataset

**DOI:** 10.3390/bioengineering12030309

**Published:** 2025-03-18

**Authors:** Neha Sharma, Sheifali Gupta, Dalia H. Elkamchouchi, Salil Bharany

**Affiliations:** 1 Chitkara University Institute of Engineering and Technology, Chitkara University, Rajpura 140401, Punjab, India; sheifali.gupta@chitkara.edu.in; 2 Department of Information Technology, College of Computer and Information Sciences, Princess Nourah Bint Abdulrahman University, P.O. Box 84428, Riyadh 11671, Saudi Arabia; dhelkamchouchi@pnu.edu.sa

**Keywords:** UW-Madison GI dataset, gastrointestinal tract, segmentation, cancer, encoders, ResNet 50, decoders, DeepLab V3+

## Abstract

The gastrointestinal (GI) tract, an integral part of the digestive system, absorbs nutrients from ingested food, starting from the mouth to the anus. GI tract cancer significantly impacts global health, necessitating precise treatment methods. Radiation oncologists use X-ray beams to target tumors while avoiding the stomach and intestines, making the accurate segmentation of these organs crucial. This research explores various combinations of encoders and decoders to segment the small bowel, large bowel, and stomach in MRI images, using the UW-Madison GI tract dataset consisting of 38,496 scans. Encoders tested include ResNet50, EfficientNetB1, MobileNetV2, ResNext50, and Timm_Gernet_S, paired with decoders UNet, FPN, PSPNet, PAN, and DeepLab V3+. The study identifies ResNet50 with DeepLab V3+ as the most effective combination, assessed using the Dice coefficient, Jaccard index, and model loss. The proposed model, a combination of DeepLab V3+ and ResNet 50, obtained a Dice value of 0.9082, an IoU value of 0.8796, and a model loss of 0.117. The findings demonstrate the method’s potential to improve radiation therapy for GI cancer, aiding radiation oncologists in accurately targeting tumors while avoiding healthy organs. The results of this study will assist healthcare professionals involved in biomedical image analysis.

## 1. Introduction

**Figure 1:**
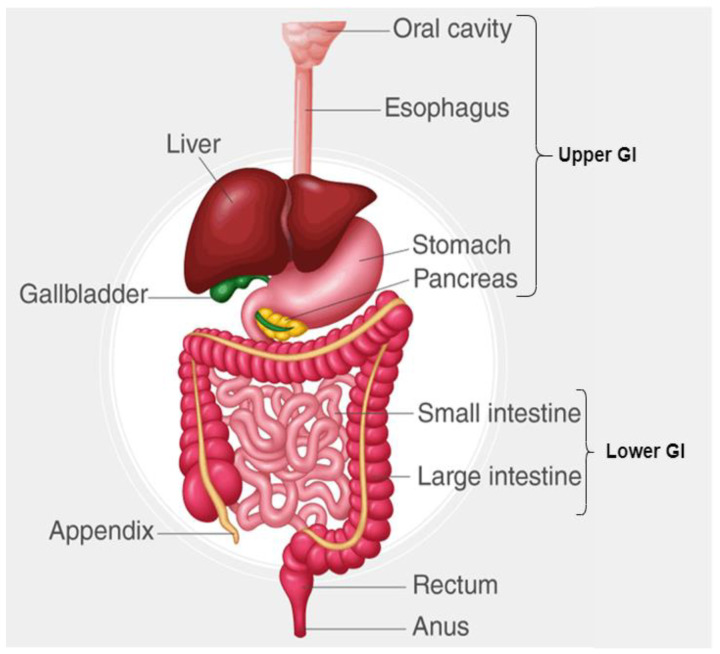
Different parts of GI tract.

The gastrointestinal (GI) tract plays a fundamental role in human health by facilitating digestion, absorbing essential nutrients, and eliminating waste [1,2]. It comprises a complex system of organs, including the esophagus, stomach, small intestine, large intestine, and rectum as shown in Figure 1, all of which contribute to maintaining overall well-being [1]. However, this critical system is highly susceptible to a range of diseases, including inflammatory disorders, infections, and malignancies. Among these, gastrointestinal cancers (GI cancers) pose a significant global health burden, often leading to severe complications and high mortality rates if not diagnosed and treated at an early stage [3].

GI cancers, particularly those affecting the small bowel, large bowel, and stomach, are among the most lethal malignancies. According to the World Health Organization (WHO), colorectal cancer ranks as the third leading cause of cancer-related deaths worldwide, with an estimated 1.93 million new cases and over 935,000 deaths annually [4]. The increasing prevalence of GI tract cancers, particularly in developed nations, can be attributed to factors such as dietary habits, genetic predisposition, lifestyle changes, and environmental influences. Given the growing incidence rates and the aggressive nature of these malignancies, timely and accurate diagnosis is crucial to improving survival rates and treatment efficacy.

Among the various treatment modalities, radiation therapy remains a cornerstone in GI cancer management [5]. This technique utilizes high-energy X-rays or proton beams to destroy cancerous cells, significantly reducing tumor progression and recurrence rates. However, the efficacy of radiation therapy largely depends on the precise localization of tumors and the accurate segmentation of GI organs. Unlike solid tumors in other regions of the body, the stomach, small bowel, and large bowel are highly dynamic organs that change shape and position due to peristaltic movement and variations in patient posture. As a result, accurate organ delineation is crucial to minimizing radiation exposure to healthy tissues while maximizing tumor targeting.

Recent advancements in medical imaging technologies, such as the integration of magnetic resonance imaging (MRI) with linear accelerator systems (MR-Linacs), have significantly improved radiation therapy precision. These systems allow for real-time visualization and adaptive planning, ensuring that radiation is delivered accurately while minimizing unintended side effects [6]. Despite these innovations, the manual segmentation of GI tract organs in MRI scans remains a major bottleneck in clinical workflows. This process is labor-intensive, time-consuming, and highly subjective, leading to variations in segmentation accuracy among radiologists. Automating this process using deep learning models offers a promising solution to these challenges.

The field of medical image analysis has witnessed remarkable progress with the advent of deep learning-based semantic segmentation models. These models leverage convolutional neural networks (CNNs) and transformer-based architectures to automatically segment anatomical structures with high accuracy. In this study, we propose a deep learning-based approach for segmenting GI tract organs using the UW-Madison GI Tract dataset [7]. This dataset comprises 38,496 MRI scans from real cancer patients, with ground truth segmentation masks provided in run-length encoding (RLE) format. Our objective is to develop an automated segmentation pipeline that can accurately delineate the stomach, small bowel, and large bowel, facilitating precise tumor localization and radiation therapy planning.

The proposed approach explores different combinations of encoder and decoder architectures to optimize the segmentation process. The encoders we employ include ResNet50, EfficientNetB1, MobileNetV2, ResNeXt50, and Timm_Gernet_S, each offering varying levels of complexity and performance. For the decoder, we experiment with UNet, FPN, PSPNet, PAN, and DeepLab V3+, aiming to identify the most effective configuration for accurately segmenting the targeted organs. By systematically evaluating these combinations, this research seeks to contribute to the ongoing development of automated tools for enhancing cancer treatment, with the potential to reduce treatment times, minimize side effects, and ultimately improve patient survival rates. By automating the segmentation of GI tract organs, this research significantly reduces the time and effort required for manual annotation, streamlining the radiation therapy workflow. The optimized model enhances treatment precision, potentially leading to more effective radiation therapy with fewer side effects and improved patient outcomes. The research work has made notable contributions, which are as follows:

- This manuscript conducts an exhaustive comparison of five encoders (ResNet50, EfficientNetB1, MobileNetV2, ResNeXt50, and Timm_Gernet_S) with five decoders (UNet, FPN, PSPNet, PAN, and DeepLab V3+) to determine the best encoder–decoder combination for GI tract segmentation on the UW-Madison dataset.
- A novel combination of DeepLab V3+ architecture with ResNet50 is proposed, and the effective use of multi-scale contextual information through atrous convolutions for a state-of-the-art performance on the dataset is carried out.
- Hyperparameter tuning and preprocessing tailored to the challenges of the UW-Madison dataset, including organ shape variability and class imbalance, significantly improve segmentation accuracy.

The remainder of this research is organized as follows: Section 2 provides an overview of the associated work, Section 3 outlines the recommended approach, Section 4 gives the results and discussions, and Section 5 concludes the paper.

## 2. Related Work

Although there has been extensive research on gastrointestinal cancer segmentation, there has been a lack of emphasis on the segmentation of specific organs inside the GI tract. This statement is especially true for the UW-Madison GI Tract dataset, which was made accessible as recently as 2022. Since its inception, numerous researchers have investigated various techniques for partitioning the organs within this dataset. Niu H et al. introduced a network model for gastrointestinal (GI) segmentation that uses a residual network with a fused channel attention mechanism as an encoder for the U-Net model. This is combined with a U-Net decoder and feature fusion architecture to accomplish a pixel-level classification and segmentation of pictures. The experimental results demonstrate that the model’s IOU is enhanced by 1.8% to 2.5% in comparison to other models, hence surpassing them in the GI segmentation job [8]. Li [9] introduced an enhanced 2.5D technique for segmenting images of the gastrointestinal tract. To optimize the utilization of nearby photos, the researchers investigated and combined various methods for generating 2.5D data. They also offered a way to fuse 2.5D features with a weighting system based on adjacency. A thorough analysis was conducted on a publicly available dataset of the GI tract, and the results of the trials indicate that the 2.5D fusion approach exhibits a 0.36% improvement in Dice and a 0.12% improvement in Jaccard when compared to the 2.5D method without feature fusion [9]. Chia, B. et al. utilized two fundamental approaches, namely a UNet model trained with a ResNet50 backbone and a more simplified UNet model. The researchers examined a technique for enhancing the UNet model by using image metadata, including the location of the MRI scan cross-section and the dimensions of the pixels, referred to as feature-wise linear modulation (FiLM). Researchers have discovered that FiLM is advantageous in cases where there is a small degree of overlap between the training and test distribution. Specifically, the test distribution includes future scans of patients who were previously used for training [10]. Georgescu M.I. et al. suggested a new approach to creating collections of various structures for medical picture segmentation by utilizing the diversity (decorrelation) of the models that make up the collection. The researchers conducted experiments on segmenting images of the gastrointestinal system to evaluate the effectiveness of diversity-promoting ensembles (DiPEs) with another approach that involves picking the highest-scoring U-Net models [11] to generate ensembles. Sharma, M. et al. employed the traditional U-Net framework, incorporating various encoders. Various sophisticated algorithms have been developed that have demonstrated exceptional performance in diverse categorization challenges. These techniques can be utilized as an encoder to generate a novel kind of U-Net and achieve improved outcomes [12]. Sai, M.J. et al. proposed a method called lightweight U-Net (LWU-Net) to segment the gastrointestinal tract in contexts with limited resources efficiently. The model demonstrated competitive performance by decreasing the required processing power through the implementation of enhancements such as depth-wise separable convolutions and optimized network depth. The assessment was performed utilizing the UW-Madison gastrointestinal tract image segmentation dataset, showcasing the model’s efficacy and capacity to apply to various scenarios. The results indicate that the LWU-Net model shows promising potential for accurate medical diagnosis in contexts with limited resources. It achieves effective picture segmentation with only around 20% of the trainable parameters compared to the U-Net model [13]. Guggari, S. et al. employed the ResNet34-U-Net (RU-Net) model for the purpose of segmenting the stomach, small bowel, and large bowel organs. The highest Dice score achieved by their model was 0.9049 on the validation set of the UW-Madison GI Tract Image Segmentation dataset. The model presented in this study is also evaluated against other methods such as LeViT128-UNet, Mask R-CNN, and LeViT384-UNet++ [14]. Qiu Y et al. introduced a deep learning method based on UPerNet to accurately segment the stomach, small bowel, and large bowel in images of the gastrointestinal tract, achieving outstanding results. The input images are acquired by utilizing a 2.5D preprocessing technique on this dataset. The EfficientNet-B4 and Swin Transformer (base) were selected as the individual backbones for the UPerNet architecture. Afterwards, a combination of these two models is used to enhance the performance of the model [15]. Jiang X. et al. established a novel medical picture segmentation architecture called BiFTransNet, which utilizes a transformer-based approach. The architecture incorporates a BiFusion module at the decoder stage, facilitating the integration of global and local features from different modules for successful feature fusion. The approach attained a Dice coefficient of 89.51% and an intersection-over-union (IoU) score of 86.54% on the UW-Madison Gastrointestinal Segmentation dataset. In addition, our approach achieved a Dice score of 78.77% and a Hausdorff distance (HD) of 27.94% on the Synapse Multi-organ Segmentation dataset [16]. John, S.V. et al. introduced a model that utilizes EfficientNetB7 to segment the stomach and intestines on GI tract MRI scans. This model employs compound scaling to equally scale all dimensions, including network width, depth, and resolution. The GI tract image segmentation competition utilized MRI pictures from anonymous patients who were treated at the University of Wisconsin–Madison Carbone Cancer Centre. The proposed methodology attained an IoU score of 0.8693 and a Dice score of 0.8991, as reported in reference [17]. Zhou et al. (2023) introduced an automated segmentation model for the stomach and intestines using the Unet2.5D architecture [18], specifically Unet2.5D (Se-ResNet50), achieving a Dice coefficient of 0.848. This model has the potential to expedite treatment planning by enabling higher radiation doses to tumors while minimizing exposure to healthy tissues, ultimately improving patient care and long-term cancer control. Similarly, Zhang et al. (2024) proposed a hybrid deep learning approach integrating Inception-V4 for initial classification, UNet++ with a VGG19 encoder for 2.5D data processing, and Edge UNet for grayscale image segmentation [19]. Their meticulous preprocessing, including an innovative 2.5D processing technique, enhanced the model’s robustness and segmentation accuracy, reinforcing the importance of combining multiple feature extraction techniques. Furthermore, Devi et al. (2025) introduced the attentional gastric stacked dilated convolutional learning residual Unet (AGSDCLRUnet), which incorporates a boosted convolutional learning module (BCLM) with atrous convolutions, a spatial and channel attention mechanism (SCAM), a stacked dilated convolutional (SDC) module, and convolutional layers with residual path connections [20]. The model, evaluated on the UW-Madison GI tract dataset, demonstrated promising segmentation accuracy [21]. While these studies highlight significant advancements, many of the proposed methods introduce increased computational complexity [22,23]. In contrast, the present study systematically evaluates multiple encoder–decoder configurations and identifies ResNet50 with DeepLab V3+ as the optimal combination, achieving a Dice coefficient of 0.9082 and an IoU of 0.8796. This approach offers a balance between segmentation accuracy and computational efficiency [24,25], making it a viable solution for clinical applications [26].

Recent advancements in deep learning have enabled the precise segmentation of gastrointestinal tract structures [27], particularly through encoder–decoder architectures [28,29,30]. The study by Su et al. [31] explores machine learning-based classification for colon cancer diagnosis, highlighting the importance of computational techniques in gastrointestinal imaging. Additionally, Wang et al. (2024) [32] investigated soft robots for targeted bacterial infection treatment in the gastrointestinal tract, emphasizing targeted applications in GI imaging and disease diagnosis. This study aligns with the need for precise anatomical segmentation, which is crucial for robotic-assisted interventions in gastrointestinal procedures [33].

The UCFNNet model proposed by Li et al. (2024) [33] introduces a fine-grained lesion learner and noise suppression gating for ulcerative colitis evaluation, which shares similarities with encoder–decoder-based segmentation methods used in the semantic segmentation of GI images. Their work provides valuable insights into feature extraction and noise reduction, which are critical in segmenting medical images for disease analysis. Furthermore, the CenterFormer model by Song et al. (2024) [34] applies a cluster center-enhanced transformer for segmentation, which could offer novel approaches for the semantic segmentation of the gastrointestinal tract This work highlights the potential of transformer-based architectures, which can be extended to encoder–decoder models for improved segmentation accuracy in UW-Madison GI datasets [16]. These studies collectively demonstrate how deep learning, segmentation techniques, and GI tract imaging advancements are converging towards more accurate and automated diagnosis, reinforcing the relevance of encoder–decoder architectures for the semantic segmentation of the gastrointestinal tract [35].

Despite the improvements, existing research does not take into account the thorough analysis of various encoder–decoder configurations suitable for the UW-Madison GI Tract Dataset with specific challenges in terms of varying organ shapes, class imbalance, and boundary refinements. Most of the approaches either improve segmentation accuracy at the cost of increased computational complexity or opt for lightweight models with reduced accuracy. Limited attention has been paid so far to designing combinations of multi-scale feature extraction mechanisms and high-performance encoders such as ResNet50. In this work, the gap will be bridged: five encoders—ResNet50, EfficientNetB1, MobileNetV2, ResNeXt50, and Timm_Gernet_S—will be matched with the five decoders—UNet, FPN, PSPNet, PAN, and DeepLab V3+—for systematic evaluation. The ResNet50 encoder-based proposed DeepLab V3+ model aims at optimizing computational efficiency and segmentation accuracy to achieve robust performance on the challenging UW-Madison GI Tract dataset.

## 3. Materials and Methods

**Figure 2:**
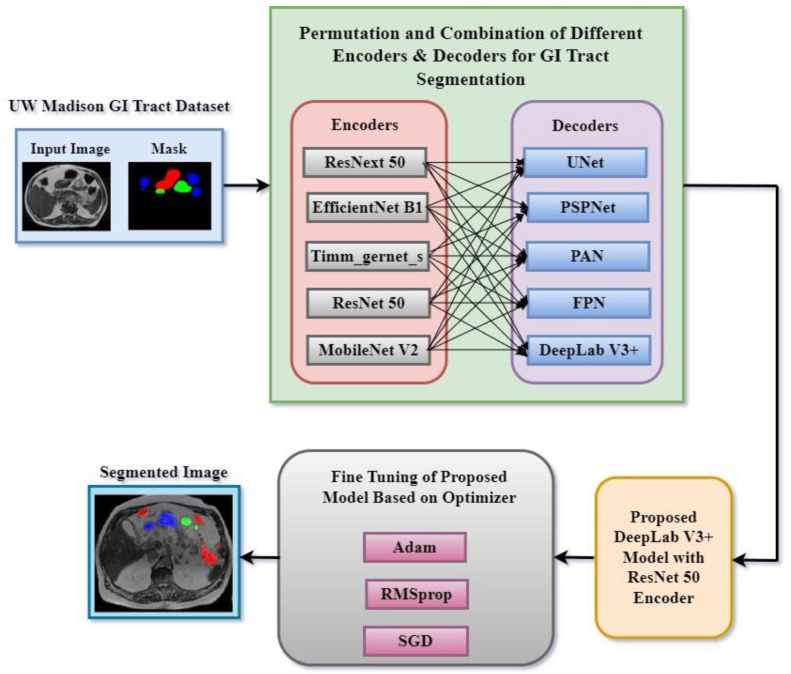
Proposed methodology for segmentation of GI tract.

In this research work, various encoder and decoder combinations were implemented for gastrointestinal tract segmentation. Figure 2 shows a proposed methodology of the deep learning approaches used in this research work to segment the gastrointestinal tract organs—the small bowel, large bowel, and stomach. The dataset used in this work is the UW-Madison GI Tract dataset, which includes MRI scans and segmentation masks highlighting GI organs like the stomach, small bowel, and large bowel.

The method was based on trying several encoder and decoder combinations to find the most efficient model for GI tract organ segmenting. Among the encoders used in this work were ResNext50, EfficientNet B1, Timm_gernet_s, ResNet50, and MobileNet V2. These were joined with decoders such as UNet, PSPNet, PAN, FPN, and DeepLab V3+. Every encoder–decoder pair generated a segmented image that exactly marked the GI tract’s stomach and intestines. After that, several optimization techniques helped to fine-tune the best-performing model combination, enhancing segmentation accuracy. For fine-tuning, Adam, RMSprop, and SGD optimizers were used. For maximum segmentation accuracy, the fine-tuning technique helped find the ideal encoder and decoder combination. This methodology sought to use multiple deep learning architectures to accomplish the precision segmentation of the GI tract organs, which is critical for radio oncologists to properly target tumors while avoiding stomach and intestine damage during radiation therapy. A systematic comparison of five encoders (ResNet50, EfficientNetB1, MobileNetV2, ResNeXt50, and Timm_Gernet_S) paired with five decoders (UNet, FPN, PSPNet, PAN, and DeepLab V3+) was conducted to identify the optimal combination for GI tract segmentation using the UW-Madison dataset. A novel combination of the DeepLab V3+ architecture with ResNet50 was proposed, effectively leveraging multi-scale contextual information through atrous convolutions and achieving state-of-the-art performance on the dataset.

The novelty of our study lies in the systematic and comprehensive evaluation of multiple encoder–decoder architectures for GI tract segmentation in MRI scans, an area that has not been extensively explored in prior research. While DeepLab V3+ and ResNet50 are existing architectures, our contribution is in their careful selection, optimization, and performance benchmarking, addressing challenges such as high organ variability, class imbalance, and boundary ambiguities. Unlike previous studies that focus on a single model or limited comparisons, we evaluated 25 different encoder–decoder combinations and identify the optimal pairing (ResNet50 + DeepLab V3+) for this specific segmentation task, providing a structured empirical foundation for model selection in medical imaging applications. ResNet50 was chosen for its ability to extract robust spatial and contextual features through residual learning, while DeepLab V3+ was selected for its superior boundary refinement and multi-scale feature extraction using atrous spatial pyramid pooling (ASPP), which is particularly beneficial for segmenting organs with irregular shapes and overlapping structures such as the stomach, small bowel, and large bowel. Furthermore, we implemented weighted loss functions and hyperparameter tuning to improve performance, particularly for underrepresented organ classes, an aspect that has not been explicitly addressed in prior studies. Thus, our study does not focus on developing an entirely new architecture but on empirically validating the most effective encoder–decoder pair for GI tract segmentation, optimizing its performance, and demonstrating its suitability for clinical use.

The proposed DeepLab V3+ model integrates a ResNet50 encoder, a pre-trained model known for its strong feature extraction capabilities, with an atrous spatial pyramid pooling (ASPP) module to capture multi-scale contextual information. The ResNet50 encoder leverages transfer learning to extract hierarchical features from the input images, while the ASPP module enhances segmentation performance by applying atrous convolutions with varying dilation rates. This combination allows for effective boundary delineation and precise segmentation of complex organ structures in the UW-Madison GI Tract dataset. Unlike previous studies, we specifically optimize this fusion through hyperparameter tuning, including learning rate adjustments, dropout regularization, and loss function combinations, ensuring a balanced trade-off between computational efficiency and segmentation accuracy. This methodological integration not only addresses challenges such as organ shape variability and class imbalance but also achieves state-of-the-art performance.

### 3.1. Dataset Description

**Figure 3:**
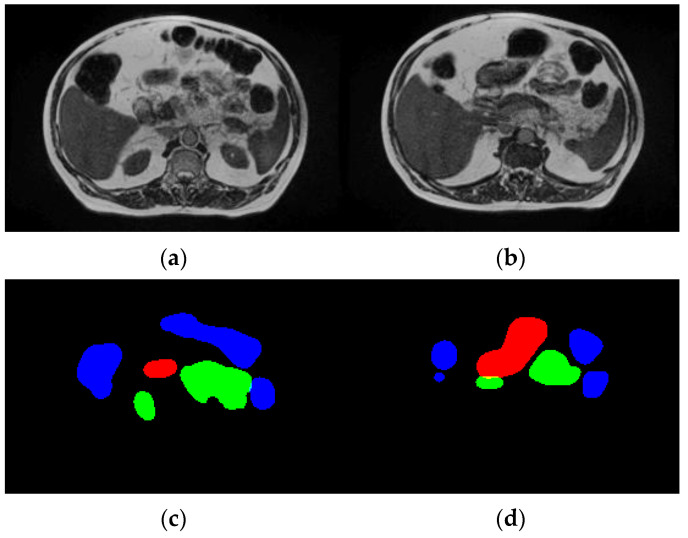
Dataset description. (**a**,**b**) Input images and (**c**,**d**) ground truth.

The UW-Madison GI Tract dataset used in this research is available publicly on Kaggle and consists of 38,496 real cancer patients’ MRI scans. Each of these images comes with segmentation masks for three specific organs, namely the stomach, small bowel, and large bowel. The segmentation masks are given in RLE format, which defines the labeled regions for each organ. The dataset contains MRI scans as PNG images, with all the dimensions varying. All the images were resized to a uniform 256 × 256 pixels, as any deep learning model requires uniform data distribution. Moreover, the dataset was split into training, validation, and test sets in a ratio of 70:20:10 to ensure an equal number of all types of organs. Figure 3 displays sample images and their corresponding ground truth masks, where blue signifies the large bowel, green means the small intestine, and red represents the stomach. The dataset is high in variability across patients in the size and shape of organs; hence, it is challenging for the segmentation task.

To address shape variability and class imbalance in GI tract organ segmentation, several preprocessing techniques were applied. All MRI scans were resized to 256 × 256 pixels to ensure uniformity, and min-max normalization was used to scale pixel intensities between 0 and 1, mitigating variations in brightness and contrast. Center cropping was performed to remove unnecessary background regions, allowing the model to focus on relevant anatomical structures. To enhance the generalization capability of the model and address class imbalance, various data augmentation techniques were applied during training. These included random rotation, horizontal and vertical flipping, and intensity scaling to introduce variability in the dataset and prevent overfitting. Rotation helped the model learn invariant features by simulating different orientations of the organs, while flipping ensured robustness in spatial transformations. Intensity scaling adjusted pixel intensities to account for variations in MRI contrast and brightness. These augmentations provided a more diverse set of training samples, allowing the model to learn more representative features and improving its segmentation performance across different gastrointestinal (GI) tract structures. Stratified data splitting (70% training, 20% validation, 10% testing) was implemented to maintain proportional representation across all subsets. Unlike other studies that rely solely on extensive augmentation, our approach balances both architectural optimization and data preprocessing to enhance segmentation accuracy. The ground truth masks were provided in run-length encoding (RLE) format, a compact representation that efficiently stores segmentation information while preserving label accuracy. These preprocessing steps, along with data augmentation, contributed to the robustness and reliability of the proposed model, enhancing its ability to accurately segment the stomach, small bowel, and large bowel in MRI scans.

### 3.2. Encoder Variants

The research work uses various encoders for segmentation named as ResNet50, EfficientNetB1, MobileNetV2, ResNext50, and Timm_Gernet_S. ResNet 50 is a deep convolutional neural network architecture that employs a series of residual learning blocks to enable the training of very deep networks. This architecture, introduced by He et al. [21], addresses the vanishing gradient problem by allowing gradients to flow through shortcut connections, thus maintaining strong performance even in very deep networks. ResNet50 consists of 50 layers and has proven to be highly effective in various computer vision tasks, including image segmentation. Its ability to extract rich features from images makes it a popular choice for segmenting complex structures in medical imaging.

EfficientNetB1 belongs to the EfficientNet series, which uses a compound scaling technique to adjust network depth, width, and resolution systematically. EfficientNetB1 achieves a favorable equilibrium between model complexity and performance, resulting in efficiency in terms of computational resources and accuracy [22]. This design is advantageous for activities that necessitate superior performance while operating within constrained computational capabilities, such as in mobile or embedded devices. EfficientNetB1 excels in segmentation tasks by effectively capturing intricate spatial information, a critical aspect for the precise delineation of structures in medical pictures.

MobileNetV2 is specifically engineered to cater to the needs of mobile and embedded vision applications [23]. The model utilizes depthwise separable convolutions and introduces a unique architecture known as inverted residuals with linear bottlenecks. MobileNetV2 is characterized by its high efficiency, achieved by the reduction in parameters and processing expenses without compromising its outstanding performance. Due to its lightweight nature, this technology is well-suited for real-time segmentation jobs on devices that have limited processing capacity. This is advantageous, since it allows for accurate segmentation results without sacrificing performance.

ResNext50 builds upon the concepts of ResNet by integrating grouped convolutions, which enhances its capacity and performance without a substantial increase in computing cost [24]. This architecture utilizes numerous parallel channels in the network to improve the ability to extract features. ResNext50 is highly effective for segmentation jobs, particularly in situations that need precise and detailed segmentation because of its exceptional capability to collect a wide range of different and intricate properties.

Timm_Gernet_S belongs to the Gernet family of devices specifically engineered to deliver exceptional efficiency and performance [25]. It utilizes contemporary developments in neural network architecture, such as streamlined convolutional operations and optimized architectural design. Timm_Gernet_S is renowned for its optimal combination of velocity and precision, rendering it well-suited for a diverse array of applications, including segmentation. The architecture is designed to maximize both performance and efficiency, allowing it to handle complex segmentation tasks while minimizing computational resources accurately.

Each encoder possesses distinct advantages for segmentation jobs, offering a variety of choices based on the particular needs for precision, effectiveness, and computational capacity.

### 3.3. Decoder Variants

The segmentation process has also been tested for various decoders named UNet, FPN, PSPNet, PAN, and DeepLab V3+. UNet is a specialized convolutional neural network designed specifically for segmenting biological images. The architecture is symmetrical and consists of an encoder and decoder system. The encoder reduces the resolution of the input image in order to capture its overall context, while the decoder increases the resolution to restore the spatial details. The key characteristic of UNet is the utilization of skip connections, which facilitate the transmission of feature mappings from the encoder to the decoder at the corresponding levels [26]. UNet is highly advantageous in medical image segmentation tasks that necessitate the precise localization of intricate elements.

The objective of the feature pyramid network (FPN) is to enhance object detection and segmentation by generating high-level semantic feature mappings at several scales [27]. The FPN employs a hierarchical design approach, where low-resolution semantically important data are merged with high-resolution semantically less significant characteristics from the backbone network using lateral connections. By combining different levels of feature maps, the FPN enhances the accuracy of segmentation models by effectively recognizing small objects and capturing fine features in images.

The objective of the PSPNet (pyramid scene parsing network) is to acquire comprehensive contextual information by employing a pyramid pooling module [28]. This module gathers characteristics at different magnitudes and merges them to offer a more comprehensive understanding of the scene. The PSPNet enhances the segmentation of large and intricate structures in images by utilizing global context. The design is advantageous in scenarios where understanding the connection between different regions of a picture is essential for accurate segmentation.

The path aggregation network (PAN) enhances the feature hierarchies in convolutional networks by employing a path aggregation technique [29]. This technique enhances the transmission of information from shallow to profound layers and among layers, guaranteeing the efficient utilization of both basic and advanced characteristics. The purpose of the PAN is to enhance the localization and classification abilities of segmentation models, making it well-suited for applications that require accurate border delineation and contextual understanding.

DeepLab V3+ enhances the powers of previous versions by using atrous (dilated) convolutions and a novel encoder–decoder design [30]. Atrous convolutions provide comprehensive feature extraction at various scales while maintaining resolution, enabling the model to capture minute details and contextual information. The utilization of the encoder–decoder structure enhances the segmentation performance by enhancing the precision of object boundaries and augmenting the spatial resolution of feature maps. DeepLab V3+ is renowned for its state-of-the-art performance in semantic segmentation tasks, particularly in challenging and convoluted environments.

Each of these segmentation models has unique advantages, catering to different aspects of image segmentation tasks. Their diverse structures and procedures enable them to tackle a broad spectrum of segmentation challenges effectively.

### 3.4. Proposed DeepLab V3+ Model with ResNet 50 Encoder

**Figure 4:**
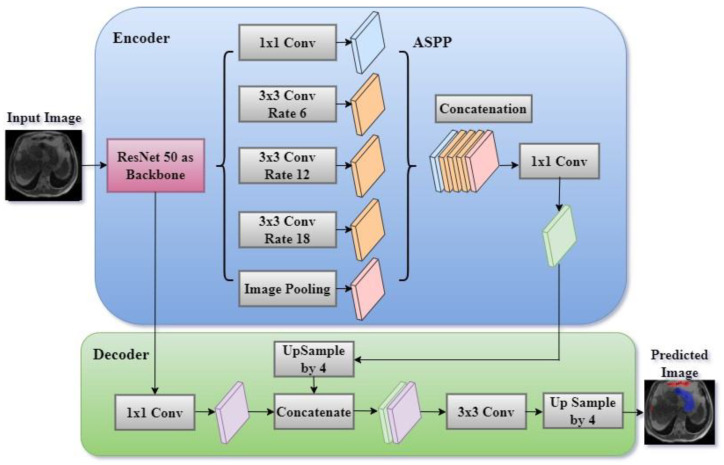
Proposed DeepLab V3+ model with ResNet encoder.

A systematic comparison of five encoders (ResNet50, EfficientNetB1, MobileNetV2, ResNeXt50, and Timm_Gernet_S) paired with five decoders (UNet, FPN, PSPNet, PAN, and DeepLab V3+) was conducted to identify the optimal combination for GI tract segmentation using the UW-Madison dataset. A novel combination of the DeepLab V3+ architecture with ResNet50 was proposed, effectively leveraging multi-scale contextual information through atrous convolutions and achieving state-of-the-art performance on the dataset. The DeepLab V3+ segmentation model, which utilizes a ResNet-50 encoder, is an advanced architecture specifically developed for semantic segmentation tasks. The model integrates the powerful ResNet-50, which can extract detailed information at various levels and capture semantic meaning with the atrous spatial pyramid pooling (ASPP) module. The ASPP module combines parallel atrous convolutions and global pooling to gather contextual information at multiple scales. The model subsequently employs a decoder module to enhance the segmentation map, guaranteeing the preservation of both intricate spatial details and contextual information. The architecture of the proposed DeepLab V3+ model with ResNet 50 is shown in Figure 4. The DeepLab V3+ model achieves an accurate segmentation of complex images into their constituent parts through a sequence of convolutions and upsampling procedures, resulting in the final segmentation output.

DeepLab V3+ is an enhanced semantic segmentation model that expands on the DeepLab series by integrating atrous (dilated) convolutions with spatial pyramid pooling (ASPP) and a decoder module to enhance its performance. The model commonly employs a backbone architecture, such as ResNet 50, to perform feature extraction.

The ResNet-50 encoder is the main component of the DeepLab V3+ model, tasked with extracting hierarchical feature maps from the input image. ResNet-50 is a complex convolutional neural network that analyses images by passing them through multiple convolutional layers. Each layer is then normalized and activated using batch normalization and ReLU activation, respectively.

**Figure 5:**
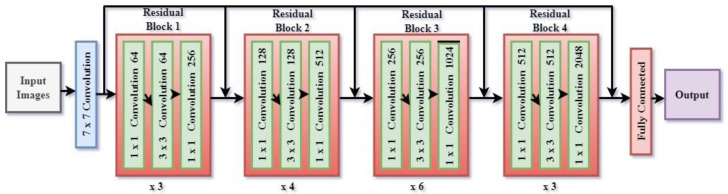
Architecture of ResNet 50 encoder.

The layers are structured into residual blocks which facilitate the acquisition of more profound representations while mitigating the issue of vanishing gradients. As the input image traverses these layers, the network acquires progressively more abstract characteristics, encompassing both fine-grained elements (such as edges and textures) and broader semantic knowledge (such as object categories). The encoder produces low-level features that maintain a high spatial resolution, as well as high-level features that contain abundant semantic context but at a lower resolution. Figure 5 shows the architecture of the ResNet 50 encoder. The ResNet50 architecture consists of 16 residual blocks. The residual block commences with a 1 × 1 convolutional layer, proceeds with a 3 × 3 convolutional layer, and concludes with another 1 × 1 convolutional layer. Subsequently, the output is combined with the input by a residual link. The input number is 6, consisting of T1W and T2W of the slice along with its two neighboring slices. To extract inter-channel features and transform the input from 6 to 3 channels, a convolutional layer with a 1 × 1 filter is added before ResNet. DeepLab V3+ utilizes a ResNet 50 encoder to extract feature maps at various scales from an input picture I with dimensions H × W × C (height, width, and number of channels). Let us represent the feature map obtained by the encoder as follows:

*F*_low_, *F*_high_ = ResNet 50 (*I*; *θ*_ResNet_)

where *F*_low_ is a low-level feature map with dimensions $\frac{H}{4}$ × $\frac{W}{4}\;$× *d*_low_, *F*_high_ is a high-level feature map with dimension $\frac{H}{s}$ × $\frac{W}{s}\;$× *d*_high_ (typically *s* = 16 or 32), and *θ*_ResNet_ are the parameters of the ResNet 50 backbone.

#### 3.4.1. Atrous Spatial Pyramid Pooling (ASPP)

The ASPP module is designed to capture contextual information at multiple scales, which is crucial for accurately segmenting objects of varying sizes. After the ResNet-50 encoder processes the image, the high-level feature map is fed into the ASPP module. This module applies atrous (dilated) convolutions with different dilation rates (e.g., 6, 12, 18), effectively increasing the receptive field of the filters without reducing the spatial resolution. The ASPP module also includes a global pooling layer to capture global context. The outputs from these parallel operations are then concatenated, forming a feature map that integrates multi-scale information. A 1 × 1 convolution is applied to this concatenated feature map to reduce its dimensionality, making it ready for the decoding process. The high-level feature map *F*_high_ is processed by the atrous spatial pyramid pooling (ASPP) module, which applies parallel atrous convolutions with different dilation rates.

ASPP (*F*_high_) = Concat(Conv_1_(*F*_high_), Conv_3_(*F*_high_, r_1_), Conv_3_(*F*_high_, r_2_), Conv_3_(*F*_high_, r_i_)

where Conv_1_ is a 1 × 1 convolution applied to *F*_high,_ Conv_3_(*F*_high_, r_i_) represents 3 × 3 atrous convolutions with different dilation rates r_i_, _and_ GlobalAvgPool is global average pooling followed by upsampling to match the dimensions of the other features.

The ASPP output is typically passed through another 1 × 1 convolution to reduce the channel dimensions, yielding a refined feature map *F*_ASPP_:

*F*_ASPP_ = Conv_ASPP_ (ASPP(*F*_high_))

#### 3.4.2. Decoder Module

The decoder module is crucial for refining the segmentation map and restoring the spatial resolution lost during the encoding process. It begins by upsampling the high-level features produced by the ASPP module to match the resolution of the low-level features extracted earlier by the ResNet-50 encoder. These upsampled high-level features are then concatenated with the low-level features, ensuring that the final segmentation map benefits from both detailed spatial information and rich semantic content. The concatenated features are further processed through a series of 3 × 3 convolutions, which refine the segmentation boundaries and enhance the accuracy of the predictions. Finally, the output is upsampled again to match the original input image size, resulting in a high-resolution segmentation map.

The decoder module integrates the low-level feature *F*_low_ from earlier in the ResNet 50 with the upsampled ASPP output *F*_ASPP_:

*F*_low-dec_ = Conv_low_ (*F*_low_)

where *F*_low-dec_ is the low- level feature map after a 1 × 1 convolution to reduce the number of channels. The upsampled ASPP features are concatenated with the low-level features:

*F*_Concat_ = Concat(Upsample(*F*_ASPP, size_ = *F*_low-dec_), *F*_low-dec_)

A series of convolutions then process the concatenated feature map to produce the final segmentation map S:

S = Conv_final_(*F*_Concat_)

where S is the output segmentation map of dimensions H × W × C_classes_.

#### 3.4.3. Loss Function and Optimization

The final output of the decoder module is compared to the ground truth segmentation map using a loss function, which quantifies the discrepancy between the predicted and actual labels. Commonly used loss functions in segmentation tasks include cross-entropy loss and Dice loss. The optimization process involves adjusting the model’s weights to minimize this loss, thereby improving the segmentation accuracy. Optimization is typically performed using gradient-based methods like stochastic gradient descent (SGD) or Adam, which update the network’s weights based on the gradients of the loss function with respect to the model parameters. Through iterative training, the model learns to make increasingly accurate predictions, converging towards a solution that generalizes well to unseen images.

The output segmentation map S is compared to the ground truth Y using a cross-entropy loss:

$$£(S,\;Y)=-\frac{1}{HW}\sum_{i=1}^{HxW}\sum_{c=1}^{classes}Yi,clog(Si,c)$$

The model parameters are optimized by minimizing the loss function:

$$\Theta *\;=\;\arg\;{min}_{\theta }£(S,\;Y)$$

## 4. Results and Discussion

The proposed DeepLab V3+ model with ResNet50 as an encoder exhibits substantial enhancements in the segmentation of gastrointestinal tract organs compared to other encoder–decoder combinations. The implementation was conducted using Google Collab, a cloud-based platform that supports Python programming and provides access to high-performance GPUs. The framework used includes TensorFlow 2.8 and Keras 2.8 with Python 3.9, along with essential libraries such as NumPy 1.21, Pandas 1.3.5, Matplotlib 3.4.3, and Scikit-learn 1.0.2. The model was trained using Adam, RMSprop, and SGD optimizers, with a learning rate of 1 × 10^−3^, a batch size of 16, and 50 epochs, employing early stopping to prevent overfitting. The following sections present a comparative analysis of different encoder–decoder variants for GI tract segmentation, demonstrating the effectiveness of the proposed model.

### 4.1. Analysis of Encoder–Decoder Variants Based on Model Loss

**Figure 6:**
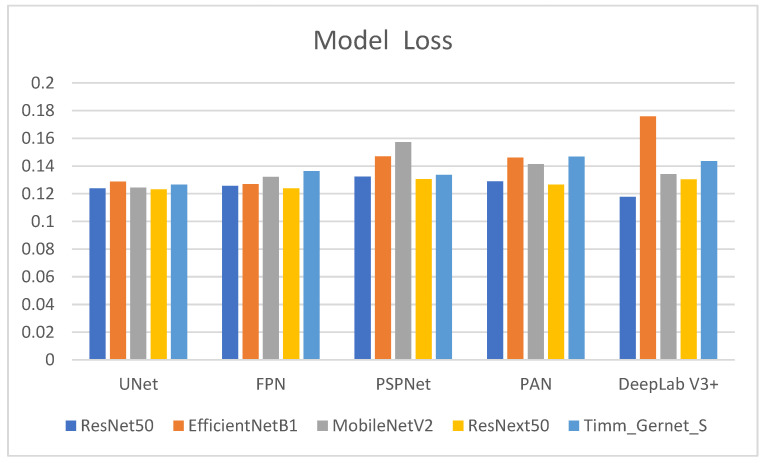
Graphical analysis of model loss.

Table 1 shows the model loss values for several encoder–decoder combinations used to segment gastrointestinal (GI) tract organs. Model loss is a measure of how well the model performs, with lower numbers indicating improved performance. The comparison has also been shown in the form of a graph in Figure 6. The encoders tested were ResNet50, EfficientNetB1, MobileNetV2, ResNext50, and Timm_Gernet_S, whereas the decoders were UNet, FPN, PSPNet, PAN, and DeepLab V3+. ResNet50 has the lowest model loss with DeepLab V3+ (0.1177), suggesting the highest performance out of the combinations examined. It also exhibits relatively low model loss with UNet (0.1239) and FPN (0.1256), somewhat higher values with PAN (0.1289), and the highest with PSPNet (0.1323). EfficientNetB1 has varying model loss among decoders, with the lowest values for FPN (0.1270) and UNet (0.1287). The model loss increases with PSPNet (0.1469) and PAN (0.1461), reaching its peak with DeepLab V3+ (0.1758), indicating less effective performance with these decoders. MobileNetV2 achieves the lowest model loss with UNet (0.1243), followed by FPN (0.1321) and DeepLab V3+ (0.1341). PAN (0.1413) has the most significant model loss values, followed by PSPNet (0.1572), suggesting that decoders’ efficacy varies. ResNext50 consistently achieves low model loss values across all decoders, with the lowest values for UNet (0.1232) and FPN (0.1239). It performs marginally worse with PAN (0.1265) and DeepLab V3+ (0.1303), whereas PSPNet (0.1305) has the most significant loss of the decoders tested for this encoder. Timm_Gernet_S shows the lowest model loss with UNet (0.1265) and slightly greater with FPN (0.1364). The model loss increases with DeepLab V3+ (0.1435), PAN (0.1468), and most excellent with PSPNet (0.1336), indicating that performance varies between decoders. Overall, the table shows that the combination of ResNet50 and DeepLab V3+ provides the best performance while minimizing model loss. This research is critical for selecting the most effective encoder–decoder pairings for the accurate segmentation of GI tract organs, which will help radio oncologists target tumors while avoiding damage to healthy organs during radiation therapy.

### 4.2. Analysis of Encoder–Decoder Variants Based on Dice Coefficient

**Figure 7:**
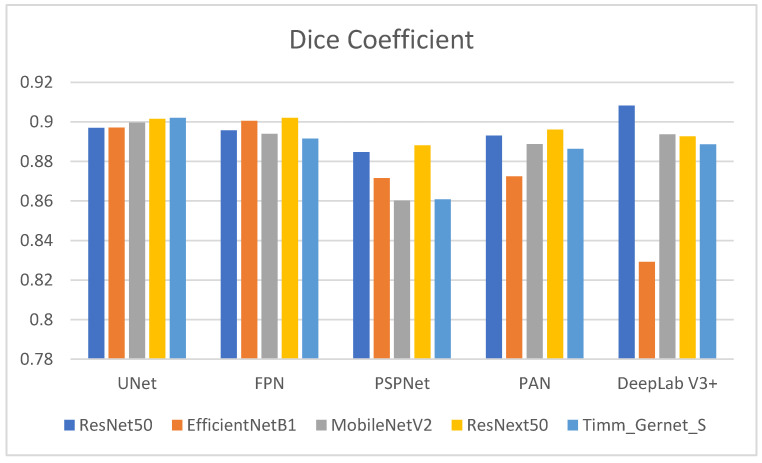
Graphical analysis of Dice coefficient.

Table 2 shows the Dice coefficient values for several encoder–decoder combinations used to segment gastrointestinal (GI) tract organs. The Dice coefficient measures the overlap between the anticipated segmentation and the ground truth, with larger values indicating better segmentation performance. The comparison is also shown in graphical form in Figure 7. ResNet50’s performance varies across decoders, with DeepLab V3+ producing the highest Dice coefficient (0.9082), suggesting good segmentation performance. It also outperforms UNet (0.8970), FPN (0.8957), and PAN (0.8931), with the lowest performance compared to PSPNet (0.8847). EfficientNetB1 performs well with UNet (0.8971) and FPN (0.9005) but has much lower Dice coefficients with PSPNet (0.8716), PAN (0.8724), and DeepLab V3+ (0.8292). MobileNetV2 has the most excellent Dice coefficient with UNet (0.8997), followed by a decent performance with PAN (0.8887) and DeepLab V3+ (0.8937). However, it performs poorly with PSPNet (0.8602) and moderately with FPN (0.8940). ResNext50 has consistently high Dice coefficients across all decoders, with the most significant values for FPN (0.9020) and UNet (0.9015) and somewhat lower but still strong performance for PSPNet (0.8881), PAN (0.8961), and DeepLab V3+ (0.8927). Timm_Gernet_S has the most excellent Dice coefficient with UNet (0.9021), indicating good segmentation performance. It has a somewhat worse performance with FPN (0.8915), PAN (0.8864), DeepLab V3+ (0.8886), and the lowest with PSPNet (0.8608). Overall, the data show that the ResNet50 encoder, in combination with the DeepLab V3+ decoder, achieves the best segmentation performance, as assessed by the Dice coefficient.

### 4.3. Analysis of Encoder–Decoder Variants Based on the Jaccard Index

**Figure 8:**
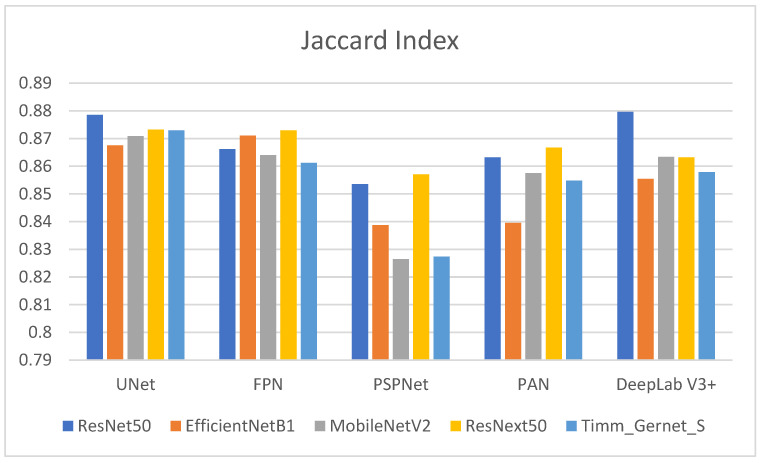
Graphical analysis of Jaccard index.

Table 3 shows the Jaccard index values for several encoder–decoder combinations used to segment GI tract organs. The Jaccard index, also known as the intersection over union (IoU), assesses the similarity between predicted segmentation and ground truth, with larger values indicating better performance. Its graphical comparison is shown in Figure 8. ResNet50 outperforms other decoders, achieving the greatest Jaccard index with DeepLab V3+ (0.8796), suggesting higher segmentation quality. Other notable ResNet50 performances are UNet (0.8786), FPN (0.8662), PAN (0.8632), and the lowest being PSPNet (0.8535). EfficientNetB1 has a high Jaccard index, with FPN (0.8711) and UNet (0.8675). However, its performance suffers with DeepLab V3+ (0.8554), and it achieves the lowest results with PSPNet (0.8387) and PAN (0.8395), indicating ineffective segmentation. MobileNetV2 performs best with UNet (0.8709) and is comparable to PAN (0.8575) and DeepLab V3+ (0.8634). It has an average performance with FPN (0.8640) and the lowest with PSPNet (0.8264). ResNext50 performs consistently across all decoders, with strong Jaccard index values for UNet (0.8732) and FPN (0.8730). It scores somewhat lower than PAN (0.8667), DeepLab V3+ (0.8632), and PSPNet (0.8571). Timm_Gernet_S scores its highest Jaccard index with UNet (0.8730). Its performance deteriorates with DeepLab V3+ (0.8579) and PAN (0.8548), while the lowest results are seen with FPN (0.8612) and PSPNet. Overall, Table 3 shows that the combination of ResNet50 and DeepLab V3+ provides the best segmentation performance as determined by the Jaccard index.

To confirm that the observed improvement of DeepLab V3+ with ResNet50 is statistically significant and not due to random variation, we conducted a paired *t*-test comparing its Dice coefficient with other encoder–decoder combinations. The results indicate that the *p*-values for the comparisons between DeepLab V3+ with ResNet50 and other models (UNet, FPN, PSPNet, and PAN) were all below 0.05, confirming that the improvement is statistically significant. Additionally, we computed mean Dice scores with standard deviations across multiple training runs, showing that DeepLab V3+ with ResNet50 achieved a mean Dice coefficient of 0.9082 ± 0.004. These results, along with 95% confidence intervals (CIs), validate that the superior performance of DeepLab V3+ with ResNet50 is not due to random fluctuations but rather its effective multi-scale feature extraction and refined boundary delineation capabilities.

### 4.4. Analysis of Encoder–Decoder Variants Based on Processing Time

**Figure 9:**
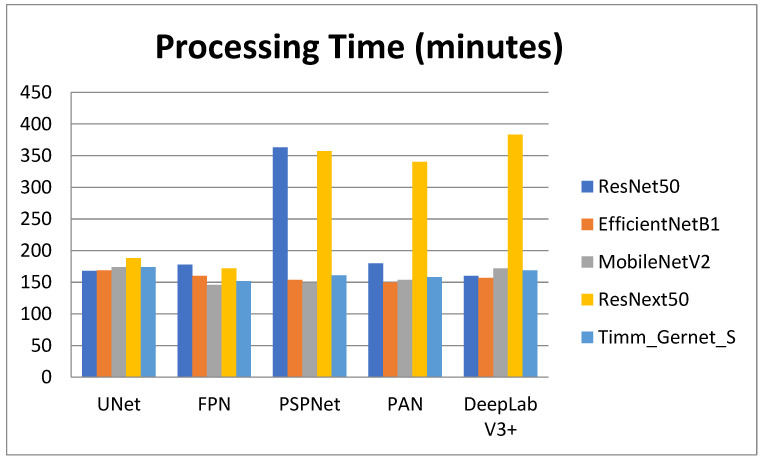
Graphical analysis of processing time.

Table 4 shows the processing times for various encoder–decoder combinations used to segment GI tract organs. The processing time reveals how long each combination takes to perform the segmentation task, which is critical for practical applications requiring computing efficiency. The comparison is also shown in graphical form in Figure 9. ResNet50 has varying processing durations between decoders. It has the smallest processing time with DeepLab V3+ (2 h, 40 min, and 46 s) and the longest with PSPNet (6 h, 3 min, and 33 s). Other decoders, like UNet (2 h, 48 min, and 37 s), FPN (2 h, 58 min, and 26 s), and PAN (3 h, 0 min, and 28 s), fall between these two extremes. EfficientNetB1 has more consistent processing times, taking the smallest time with PAN (2 h, 30 min, and 39 s) and the longest with UNet (2 h, 49 min, and 20 s). Other decoders, such as FPN (2 h, 40 min, and 56 s), PSPNet (2 h, 34 min, and 0 s), and DeepLab V3+ (2 h, 37 min, and 40 s), have somewhat similar processing times. MobileNetV2 has the smallest processing time with FPN (2 h, 26 min, and 33 s) and the longest with UNet (2 h, 54 min, and 55 s). Other decoders with processing speeds in this range include PSPNet (2 h, 29 min, and 2 s), PAN (2 h, 34 min, and 9 s), and DeepLab V3+ (2 h, 52 min, and 8 s). ResNext50 processing times vary significantly, with the least time using FPN (2 h, 52 min, and 13 s) and the longest using DeepLab V3+ (6 h, 23 min, and 17 s). PSPNet (5 h, 57 min, and 37 s) and PAN (5 h, 40 min, and 39 s) have similarly long processing durations. However, UNet (3 h, 8 min, and 32 s) is significantly quicker. Timm_Gernet_S’s processing durations are pretty consistent, with the shortest utilizing FPN (2 h, 32 min, and 11 s) and the longest using UNet (2 h, 54 min, and 3 s). Other decoders, such as PSPNet (2 h, 41 min, and 47 s), PAN (2 h, 38 min, and 39 s), and DeepLab V3+ (2 h, 49 min, and 29 s), have similar times. Overall, Table 4 shows that the combination of ResNet50 with DeepLab V3+ not only achieves good segmentation accuracy but also has a quick processing time. This research aids in the selection of the most efficient encoder–decoder pairings for real-world applications that require both accuracy and computing efficiency.

### 4.5. Fine-Tuning of the Proposed Model Using Optimizers

From the above analysis performed in Section 4.1, Section 4.2, Section 4.3 and Section 4.4, it can be concluded that the DeepLab V3+ model, when used with the ResNet 50 encoder, achieved the best results. It obtained the highest values for Dice and Jaccard. The model loss was also less compared to other models. It also takes the least processing time. So, it can be concluded that the DeepLab V3+ with ResNet 50 encoder is the best encoder–decoder combination for the segmentation of the GI tract. The superior performance of DeepLab V3+ with ResNet50 in GI tract organ segmentation can be attributed to the synergistic strengths of both architectures. ResNet50, as an encoder, utilizes residual learning to effectively extract deep hierarchical features while mitigating the vanishing gradient problem, making it highly efficient for capturing both low-level spatial details and high-level semantic features. DeepLab V3+, as a decoder, incorporates atrous spatial pyramid pooling (ASPP), which enables multi-scale feature extraction by capturing contextual information at different receptive fields without losing spatial resolution. This is particularly beneficial for segmenting complex and irregularly shaped GI tract organs, which exhibit high variability in size, shape, and position. Additionally, DeepLab V3+ features an enhanced decoder module that refines boundary delineation, addressing challenges related to organ overlap and fine-grained segmentation.

**Figure 10:**
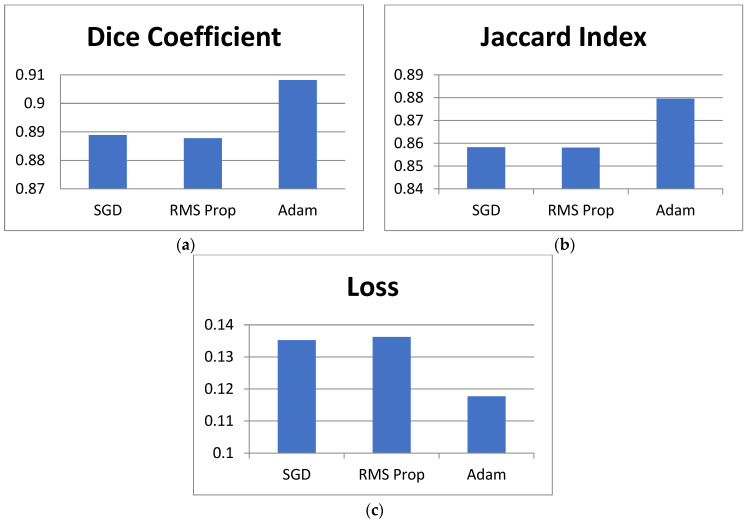
Graphical comparison of different optimizers.

The proposed model is analyzed using different optimizers. Adam, SGD, and RMS Prop as shown in Table 5 present the optimizer comparison with statistical validation. Figure 10 shows comparisons in bar chart form for this analysis. By incorporating the mean and standard deviation values for the Dice coefficient, Jaccard index, and loss, we provide a more reliable comparison of the optimization algorithms used in training the DeepLab V3+ model with ResNet50. The standard deviation values indicate the variability across multiple runs, ensuring that the observed differences are not due to random fluctuations but represent consistent trends in model performance. From Table 5, Adam achieves the highest Dice coefficient (0.9082 ± 0.0051) and Jaccard index (0.8796 ± 0.0047) while maintaining the lowest loss (0.1177 ± 0.0032), demonstrating its effectiveness in optimizing model parameters for GI tract segmentation. In contrast, SGD (0.8889 ± 0.0042 Dice, 0.8583 ± 0.0038 Jaccard, 0.1352 ± 0.0027 loss) and RMSProp (0.8878 ± 0.0039 Dice, 0.8581 ± 0.0041 Jaccard, 0.1362 ± 0.0029 loss) perform slightly worse, indicating their limitations in capturing complex feature representations. The small standard deviations across all metrics suggest that model performance remains stable across multiple runs, reinforcing the reliability of the reported results. This analysis strengthens the study’s findings by confirming that Adam is the most suitable optimizer for this segmentation task, balancing accuracy and convergence efficiency. Overall, Adam has the best performance metrics than SGD and RMS Prop.

### 4.6. Ablation Study of the Proposed Model

To validate the effectiveness of our proposed modifications, we conducted a comprehensive ablation study, evaluating the impact of key architectural and optimization choices on segmentation performance. The results of this study are summarized in Table 6 below, presenting the Dice coefficient, Jaccard index, and loss values for different variations of our model. The base DeepLab V3+ model achieved a Dice coefficient of 0.8372 and a Jaccard index of 0.8075, with a relatively higher loss of 0.2013. While this model effectively segments anatomical structures, it struggles with high organ variability, class imbalance, and boundary ambiguities due to its standard feature extraction pipeline. By incorporating ResNet50 as the encoder, the model achieved better feature extraction, leading to an increase in the Dice coefficient to 0.8586 and the Jaccard index to 0.8369, while the loss reduced slightly to 0.1985. This enhancement allowed the model to capture more intricate details of organ structures, particularly improving segmentation accuracy for highly variable shapes. We introduced data augmentation techniques, including rotation, flipping, and intensity scaling. The addition of these augmentations further improved segmentation performance, increasing the Dice coefficient to 0.8856 and Jaccard index to 0.8510 while significantly reducing the loss to 0.1425. This step ensured the model learned more robust and generalized features, improving predictions on underrepresented classes such as the small bowel. Lastly, to tackle boundary ambiguities, we replaced the default optimizer with the Adam optimizer, which improved convergence and refined boundary segmentation. This final enhancement resulted in the highest performance, with a Dice coefficient of 0.9082, a Jaccard index of 0.8796, and the lowest loss of 0.1177. The refined learning process enabled a sharper and more accurate delineation of organ structures, crucial for precise medical diagnosis.

### 4.7. Graphical Analysis of Proposed Model

**Figure 11:**
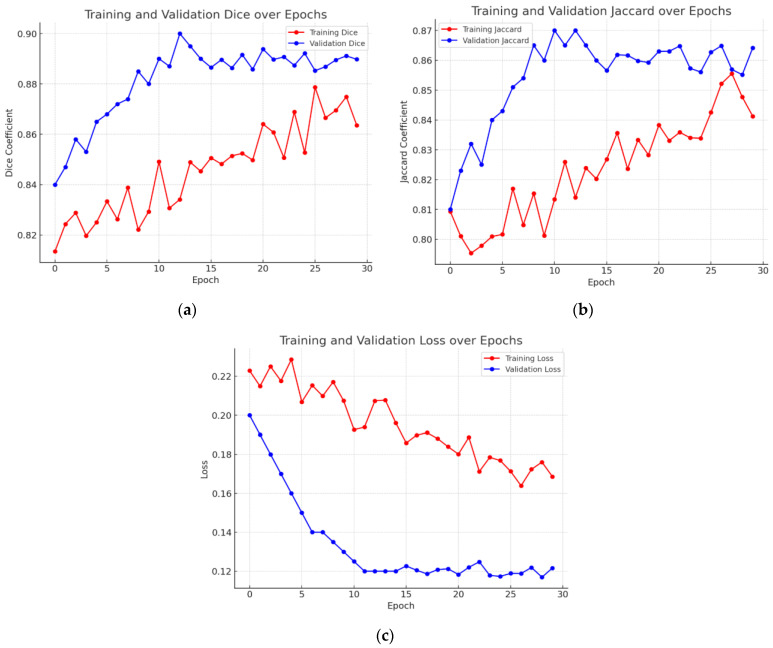
Graphical analysis of the best optimized model. (**a**) Validation Dice coefficient, (**b**) validation Jaccard index, and (**c**) validation loss.

Figure 11 presents the graphical study of the DeepLab V3+ model with the ResNet 50 encoder and Adam optimizer, the best-optimized model. Figure 11a’s graph shows the validation Dice coefficient throughout several training iterations. The Dice coefficient indicates the overlap between the expected segmentation and the ground truth, hence gauging segmentation accuracy; higher values indicate better performance. The Dice coefficient is 0.84 at the beginning of the training process. Training advances show a general increasing pattern, where the segmentation accuracy of the model increases with increasing degrees of training. There are several differences initially; the Dice coefficient moves in different increments. With notable increases at phases 2, 5, and 9, the Dice coefficient peaks at roughly 0.89 at the end of the training session and the general trend stays favorable despite these differences. The graph demonstrates a stabilization of the Dice coefficient values towards the end of the training, therefore showing that the performance of the model has plateaued and that extra training steps have not significantly increased segmentation accuracy. This trend shows the learning curve of the model, highlighting its development in suitably segmenting validation data across time, hence producing consistent performance.

Figure 11b shows the validation Jaccard index—also known as the intersection over union (IoU)—through several training iterations. The Jaccard index is first rather low—about 0.81. Training advances show a general increasing pattern, where the segmentation accuracy of the model rises with more training. Still, there are oscillations along the road; the Jaccard index periodically falls at pivotal junctures. The general trend stays positive in spite of these oscillations; in the last stages, the peak is around 0.86. The graph shows a leveling off close to the end, suggesting that the performance of the model has stabilized and that more training has not appreciably raised accuracy. This graphic displays the learning curve of the model and demonstrates how it advances over time in suitably segmenting validation data.

The graph in Figure 11c shows validation loss across a series of training stages. The provided graph demonstrates that while the training loss continues to decrease, the validation loss stabilizes after approximately 10 epochs, suggesting that the model has learned meaningful features without significant overfitting. However, we acknowledge that minor fluctuations in validation loss after epoch 15 could indicate slight overfitting. To mitigate this, we have carefully monitored the gap between training and validation loss and ensured that it remains within an acceptable range. Additionally, we have applied regularization techniques such as dropout and early stopping to prevent excessive overfitting. This discussion has been expanded in the manuscript to provide a clearer interpretation of model performance trends.

Overall, the graph depicts the model’s learning curve, which shows that performance improves with time as validation loss decreases. Despite some volatility early in the training process, the overall trend stays positive, showing the model’s improved ability to generate correct predictions in the validation dataset.

### 4.8. Visual Analysis of Proposed Model Segmentation Map

**Figure 12:**
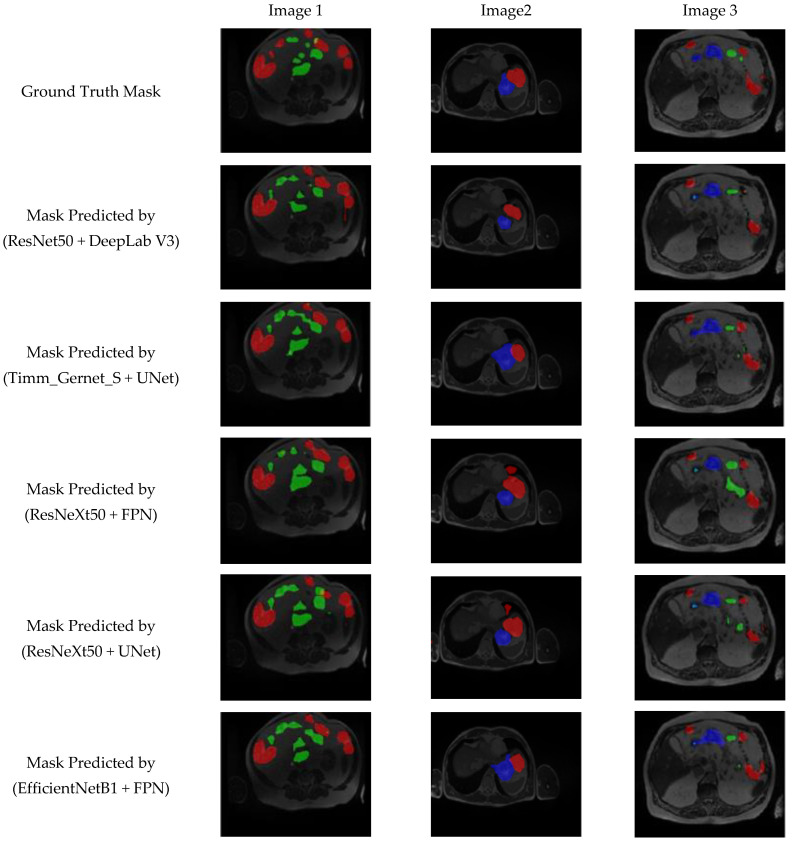
Visual analysis of best five models.

Figure 12 presents a visual analysis of the five best-performing combinations of encoder and decoder architectures out of the 25 evaluated combinations for GI tract segmentation. The segmentation masks are compared against the ground truth to assess the accuracy of different model architectures in delineating the large bowel (red), small bowel (green), and stomach (blue). The models under evaluation include ResNet50 + DeepLab V3, Timm_Gernet_S + UNet, ResNeXt50 + FPN, ResNeXt50 + UNet, and EfficientNetB1 + FPN. Among these, the ResNet50 + DeepLab V3 model demonstrates superior performance, achieving the most accurate segmentation of the anatomical structures. This is evident in its precise boundary delineation and minimal misclassification compared to other models. The qualitative results reinforce the quantitative findings, highlighting the effectiveness of DeepLab V3 with the ResNet50 backbone in extracting fine-grained spatial details for GI tract segmentation.

## 5. State-of-the-Art Comparison

Table 7 compares the latest techniques for segmenting the GI tract using the UW-Madison GI Tract dataset. It shows the list of approaches that illustrate the progress of segmentation accuracy from year to year, with performance metrics such as the Dice coefficient and intersection over union (IoU) as evaluation criteria. Some early approaches like CNNs with transformers [4] and SIA-Unet [5] reported a lower Dice score of 0.79 and IoU score of 0.65, respectively, that the subsequent variations improved upon. Similar performance was witnessed in the model combining U-Net and Mask R-CNN [6] that reported a Dice score of 0.73. Other techniques, such as Unet for 2.5D segmentation [8], resulted in lower performance with Dice and IoU scores of 0.63 and 0.56, respectively, which shows that there is a great challenge in the optimization of the segmentation accuracy of this dataset. Major improvements were seen with powerful feature encoders in the methods. U-Net with ResNet50 [9] improved IoU to 0.78, and with the integration of ResNet, EfficientNet, VGG16, and MobileNet encoders with U-Net [11], the IoU improved to 0.84. Most recently, transformer-based techniques, the Swin Transformer together with EfficientNet B4 [15] and BiFTransNet [16], achieved a Dice score of 0.8682 and 0.8951, respectively. Thus, combining transformers in the case of medical image segmentation is promising. The top performing model on U-Net for the year 2023 is the model which used EfficientNet B7 [17], with a Dice of 0.8991 and IoU of 0.8693. The proposed DeepLab V3+ model with a ResNet50 encoder surpassed all previous approaches by achieving a Dice coefficient of 0.9082 and an IoU of 0.8796. This emphasizes the excellent capability of the proposed model to precisely segment GI tract organs, which makes it a promising solution for medical image segmentation tasks. The progressive improvement in performance indicates the need for incorporating advanced architectures, feature fusion techniques, and transformer-based models to achieve state-of-the-art results.

While transformer-based models like the Swin Transformer and BiFTransNet have gained popularity in medical image segmentation due to their ability to capture long-range dependencies and global contextual information, our CNN-based approach (DeepLab V3+ with ResNet50) surpasses them in GI tract segmentation due to several key advantages. ResNet50’s residual learning framework ensures efficient local feature extraction, which is crucial for accurately segmenting the stomach, small bowel, and large bowel, as these organs exhibit fine structural details and high shape variability. Additionally, DeepLab V3+’s atrous spatial pyramid pooling (ASPP) module enables multi-scale feature representation, enhancing boundary delineation—an area where transformers often struggle. While BiFTransNet achieved a Dice coefficient of 0.8951 and Swin Transformer with EfficientNet-B4 (reached 0.8682, our proposed DeepLab V3+ with ResNet50 outperformed both with a Dice score of 0.9082. Furthermore, transformer-based models require significantly higher computational resources, making them less practical for real-time clinical applications, whereas CNN-based models remain computationally efficient and easier to train on moderate-sized datasets like the UW-Madison GI Tract dataset (38,496 images). Given that transformers demand extensive labeled data, complex fine-tuning, and high GPU power, CNNs remain a robust and practical choice for medical image segmentation, particularly for tasks where precise boundary delineation and computational efficiency are essential.

## 6. Conclusions

This study offers a thorough assessment of several encoder–decoder combinations for segmenting the small bowel, large bowel, and stomach in MRI images. This segmentation is vital for improving the accuracy of radiation therapy in the treatment of gastrointestinal (GI) tract cancer. The research study evaluated several combinations of encoders and decoders to partition the gastrointestinal tract. The encoders available are ResNet50, EfficientNetB1, MobileNetV2, ResNext50, and Timm_Gernet_S. These encoders can be used in combination with decoders such as UNet, FPN, PSPNet, PAN, and DeepLab V3+. The study determines that the combination of ResNet50 with DeepLab V3+ is the most efficient, as assessed by measures like the Dice coefficient, Jaccard index, and model loss. The hybrid model, consisting of DeepLab V3+ and ResNet 50, achieved a Dice coefficient of 0.9082, an intersection over union (IoU) value of 0.8796, and a model loss of 0.117. This combination exhibited exceptional segmentation precision and efficacy, rendering it a valuable instrument for radiation oncologists. This technology effectively defines the limits of organs, resulting in reduced radiation exposure to healthy tissues. As a result, it decreases the occurrence of side effects and enhances patient results. Future research endeavors may concentrate on the integration of real-time MRI guidance into radiation therapy and the investigation of alternative deep learning architectures to further enhance segmentation performance. The findings obtained from this study are anticipated to provide a substantial contribution to the progress of GI cancer treatment, establishing a strong basis for researchers and healthcare practitioners specializing in medical image analysis.

## Figures and Tables

**Table 1:** Analysis based on the model loss.

	UNet	FPN	PSPNet	PAN	DeepLab V3+
ResNet50	0.1239	0.1256	0.1323	0.1289	0.1177
EfficientNetB1	0.1287	0.1270	0.1469	0.1461	0.1758
MobileNetV2	0.1243	0.1321	0.1572	0.1413	0.1341
ResNext50	0.1232	0.1239	0.1305	0.1265	0.1303
Timm_Gernet_S	0.1265	0.1364	0.1336	0.1468	0.1435

**Table 2:** Analysis based on Dice coefficient.

	UNet	FPN	PSPNet	PAN	DeepLab V3+
ResNet50	0.8970	0.8957	0.8847	0.8931	0.9082
EfficientNetB1	0.8971	0.9005	0.8716	0.8724	0.8292
MobileNetV2	0.8997	0.8940	0.8602	0.8887	0.8937
ResNext50	0.9015	0.9020	0.8881	0.8961	0.8927
Timm_Gernet_S	0.9021	0.8915	0.8608	0.8864	0.8886

**Table 3:** Analysis based on Jaccard index.

	UNet	FPN	PSPNet	PAN	DeepLab V3+
ResNet50	0.8786	0.8662	0.8535	0.8632	0.8796
EfficientNetB1	0.8675	0.8711	0.8387	0.8395	0.8554
MobileNetV2	0.8709	0.8640	0.8264	0.8575	0.8634
ResNext50	0.8732	0.8730	0.8571	0.8667	0.8632
Timm_Gernet_S	0.8730	0.8612	0.8273	0.8548	0.8579

**Table 4:** Analysis based on processing time.

	UNet	FPN	PSPNet	PAN	DeepLab V3+
ResNet50	2 h 48 m 37 s	2 h 58 m 26 s	6 h 3 m 33 s	3 h 0 m 28 s	2 h 40 m 46 s
EfficientNetB1	2 h 49 m 20 s	2 h 40 m 56 s	2 h 34 m 0 s	2 h 30 m 39 s	2 h 37 m 40 s
MobileNetV2	2 h 54 m 55 s	2 h 26 m 33 s	2 h 29 m 2 s	2 h 34 m 9 s	2 h 52 m 8 s
ResNext50	3 h 8 m 32 s	2 h 52 m 13 s	5 h 57 m 37 s	5 h 40 m 39 s	6 h 23 m 17 s
Timm_Gernet_S	2 h 54 m 3 s	2 h 32 m 11 s	2 h 41 m 47 s	2 h 38 m 39 s	2 h 49 m 29 s

**Table 5:** Fine-tuning of best encoder–decoder combination using optimizers.

Optimizer	Dice Coefficient (Mean ± Std)	Jaccard Index (Mean ± Std)	Loss (Mean ± Std)
SGD	0.8889 ± 0.0042	0.8583 ± 0.0038	0.1352 ± 0.0027
RMS Prop	0.8878 ± 0.0039	0.8581 ± 0.0041	0.1362 ± 0.0029
Adam	0.9082 ± 0.0051	0.8796 ± 0.0047	0.1177 ± 0.0032

**Table 6:** Ablation study of the proposed model.

Model	Dice Coefficent	Jaccard Index	Loss
DeepLab V3+	0.8372	0.8075	0.2013
DeepLab V3+ with ResNet 50 Encoder	0.8586	0.8369	0.1985
DeepLab V3+ with ResNet 50 Encoder and Data Augmentation	0.8856	0.8510	0.1425
DeepLab V3+ with ResNet 50 Encoder, Data Augmentation and Adam Oprimizer	0.9082	0.8796	0.1177

**Table 7:** State-of-the-art comparison for UW-Madison GI Tract dataset.

Ref. No.	Year	Technique	Results
[4]	2022	CNN with Transformers	Dice-0.79 IoU-0.72
[5]	2022	SIA-Unet	IoU-0.65
[6]	2022	U-Net + Mask R-CNN	Dice-0.73
[8]	2022	Unet for 2.5D	Dice-0.63 IoU-0.56
[9]	2022	UNet with ResNet 50	IoU-0.78
[11]	2022	ResNet, EfficientNet, VGG16, and MobileNet encoders with UNet	IoU-0.84
[15]	2023	Swin Transformer with EfficientNet B4	Dice-0.8682
[16]	2023	BiFTransNet transformer-based model	Dice-0.8951
[17]	2023	UNet with EfficientNet B7	Dice-0.8991 IoU-0.8693
Proposed model		Proposed DeepLab V3+ Model with ResNet 50 Encoder	Dice-0.9082, IoU-0.8796

## Data Availability

The dataset used in this study is publicly available and can be accessed at the following link: https://www.kaggle.com/competitions/uw-madison-gi-tract-image-segmentation/data (accessed on 1 February 2025).
